# Discharging Patients With Pending Microbiology Cultures: A Narrative Review of Safety and Best Practices

**DOI:** 10.7759/cureus.84905

**Published:** 2025-05-27

**Authors:** George Bechir, Angelina Bechir

**Affiliations:** 1 Hospital Medicine, Franciscan Health, Munster, USA; 2 Genetics, Clemson University, South Carolina, Clemson, USA

**Keywords:** antibiotic stewardship, discharge communication, electronic medical records, hospital discharge safety, microbiology follow-up, patient safety, pending culture results, post-discharge care, quality improvement, test result management

## Abstract

Discharging patients with pending microbiology culture results from the hospital has become a common practice aimed at improving hospital throughput and reducing length of stay. One of the main contributors to prolonged hospitalization is the delay in receiving finalized culture results, especially when patients are clinically stable and ready for discharge. Consequently, many hospitals have explored strategies to safely discharge patients before culture results are finalized. While this approach may help alleviate bed shortages and reduce healthcare costs, it raises important patient safety concerns. Finalized culture results may reveal pathogens or resistance patterns that necessitate changes in antimicrobial therapy. Without timely review and action, these findings can lead to missed diagnoses, suboptimal treatment, or avoidable readmissions.

This narrative review synthesizes the current evidence on the safety, clinical impact, and system-level strategies associated with discharging patients with pending culture results. Studies show that 2%-11% of pending cultures in hospitalized patients require a change in clinical management, yet documentation of these pending results is often missing from discharge summaries, and follow-up responsibility is frequently unclear. Vulnerable populations, such as older adults and those discharged to subacute care facilities, are particularly at risk due to fragmented transitions and limited outpatient monitoring. Adverse outcomes in these groups include delayed therapy adjustments, inappropriate antimicrobial use, and increased healthcare utilization.

To address these risks, several interventions have shown promise. These include electronic health record (EHR)-based alerts, pharmacist-led stewardship programs, and auto-populated discharge summary tools. When integrated into standardized discharge workflows and supported by institutional policy, these interventions improve follow-up rates and reduce harm. Systematic reviews of hospital discharge practices emphasize that multidisciplinary approaches, combining clinical teams, pharmacists, and care transition services, are most effective in ensuring timely review and response to finalized results.

In conclusion, discharging patients with pending microbiology cultures can be safe when supported by structured systems that ensure results are reviewed and acted upon after discharge. Hospitals should implement protocols that clearly document pending tests, assign follow-up responsibility, and utilize EHR tools to facilitate communication. These strategies enhance both patient safety and operational efficiency during the vulnerable transition from inpatient to outpatient care.

## Introduction and background

Hospital discharge represents a vulnerable transition in care, during which lapses in communication and incomplete workups can lead to adverse outcomes [1]. One increasingly common but understudied practice is discharging patients with pending microbiology culture results [2,3]. While early discharge promotes hospital efficiency, it also introduces safety risks if finalized results later reveal pathogens or resistance patterns requiring treatment modifications [3,4]. Without structured follow-up systems, these results may go unreviewed, resulting in missed diagnoses or therapy delays [2,3,5].

As hospitals aim to reduce unnecessary inpatient days, interest is growing in whether it is safe to discharge clinically stable patients with pending test results. However, doing so without systems to track and act on those results can compromise patient safety. This narrative review synthesizes current evidence on the prevalence, risks, and potential solutions related to discharging patients with pending microbiology cultures. By identifying patterns, outcomes, and interventions, we aim to inform safer discharge practices and support the development of effective safeguards.

## Review

Methods

This narrative review was conducted to synthesize existing literature on the safety, outcomes, and best practices associated with discharging patients with pending microbiology culture results. Relevant studies were identified through a targeted search of PubMed, Google Scholar, and relevant institutional databases using keywords such as "pending cultures," "hospital discharge," "follow-up of test results," and "microbiology results post-discharge." Sources included peer-reviewed original research, systematic reviews, quality improvement studies, and relevant dissertations published between 2000 and 2024. Studies were selected based on their relevance to discharge practices, patient outcomes, documentation, or follow-up interventions. Findings were grouped thematically to highlight prevalence, risks, and proposed solutions.

Prevalence and risk of missed results

Discharging patients with pending microbiology cultures is common and may introduce avoidable clinical risk. Roy et al. found that 41% of patients were discharged with at least one pending test result, 9.4% of which were deemed clinically actionable. Notably, over 60% of discharging physicians were unaware of these results, and more than 12% required urgent follow-up [1].

El-Kareh et al. reviewed 77,349 inpatient microbiology results and found that 11% of positive cultures were finalized after discharge. Of those, 4.2% were clinically important and had not been addressed at the time of discharge. Manual review revealed that more than half likely required a change in antimicrobial therapy, most commonly involving urine and wound cultures [2].

Elderly patients transitioning to subacute care may be at greater risk due to limited communication and follow-up mechanisms. In a detailed analysis of Medicare patients, Walz reported that more than 27% were discharged with pending blood, urine, or sputum cultures, and those with preliminary results had higher rates of emergency department visits and infection-related readmissions [3]. While this source is a dissertation and not peer-reviewed, it remains one of the most comprehensive studies focused on this high-risk population.

In a related peer-reviewed study, Walz et al. found that only 11% of pending laboratory tests were documented in discharge summaries for patients discharged to subacute care facilities [4]. This documentation gap may contribute to missed follow-ups and incomplete treatment.

Kailani et al. presented a conference abstract (non-peer-reviewed) describing a pharmacist-led stewardship initiative at Dartmouth-Hitchcock in which finalized cultures were reviewed daily post-discharge. The program achieved 100% review of results within 72 hours, and 3.5% of patients required a change in therapy [6].

A systematic review by Darragh et al. indicated that approximately 30%-40% of tests pending at discharge return potentially actionable results that could necessitate changes in patient management, highlighting the critical need for timely follow-up [7].

Clinical consequences of missed cultures

Failure to act on finalized culture results after discharge can lead to delays in treatment, inappropriate antimicrobial use, and preventable readmissions. Roy et al. highlighted multiple cases in which finalized blood or wound cultures indicated the need for treatment changes, yet these results went unnoticed by the treating team post-discharge [1].

A study by Whitehead et al. emphasized that failure to follow up on microbiology results pending at discharge can delay diagnosis and treatment of significant infections, potentially harming patients and increasing the risk of litigation [8].

Fay et al. demonstrated similar risk in the outpatient setting. In a pharmacist-led stewardship program at an urgent care clinic, 11% of discharged patients were flagged for follow-up based on pending cultures, and among those, 23% required a new prescription based on finalized results [9]. Without a structured review, these patients would have continued on incorrect or suboptimal therapy.

Documentation and responsibility gaps

One of the primary challenges in safely discharging patients with pending cultures is the lack of clear documentation and follow-up ownership. Walz et al. found that only 11% of pending tests were recorded in discharge documentation for subacute care patients [4]. In another study, Kantor et al. found that only 18% of pending results were documented in discharge summaries before the implementation of an EMR-integrated tool; this improved to 43% post-intervention [5].

Beyond documentation, responsibility for follow-up is often ambiguous. Roy et al. reported that in approximately one-third of cases involving actionable results, no provider was aware that the test had been ordered or finalized [1]. This lack of clarity leaves patients vulnerable to missed treatment changes, especially when care transitions across teams or care settings.

Effective interventions

Electronic Alerts

Electronic health record (EHR)-based alert systems may improve clinician awareness of pending results. El-Kareh et al. showed that targeted email alerts for finalized microbiology results increased documented follow-up from 13% to 28% (p = 0.01) [2]. Darragh et al., in a systematic review, found that while such alerts can be effective, they must be tied to defined workflows and assigned roles to prevent alert fatigue and ensure accountability [7].

Discharge Summary Tools

Kantor et al. introduced an auto-populated discharge summary tool that significantly increased documentation of pending studies from 18% to 43% [5]. However, Whitehead et al. concluded that documentation tools alone are insufficient unless paired with education, policy support, and integration into standard discharge workflow [8].

Pharmacist-Led Review

Van Abel et al. demonstrated that a structured post-discharge microbiology result review program led by infectious diseases pharmacists resulted in timely intervention for over 29% of patients and improved coordination of follow-up care [10].

Fay et al. reported that in an urgent care setting, a similar pharmacist-led program led to improved antibiotic prescribing and reduced the need for reactive follow-up after discharge [9].

Multidisciplinary and System-Level Approaches

Darragh et al. [7] and Whitehead et al. [8] both emphasized that no single intervention is likely to be sufficient in isolation. The most effective strategies combined EHR-based tools, stewardship pharmacist involvement, standardized documentation practices, and clearly assigned follow-up responsibility. Institutional support and workflow integration are critical to success. Communication lapses during care transitions, including uncoordinated microbiology results and medication discrepancies, have also been shown to increase the risk of adverse events among older adults [11].

## Conclusions

Discharging patients with pending microbiology culture results is a common practice in hospital medicine, often driven by efforts to reduce length of stay and improve bed availability. However, this approach can lead to serious clinical consequences if finalized results are not reviewed or acted upon. Studies consistently show that a meaningful proportion of pending cultures, particularly blood, urine, and wound, require post-discharge intervention. Yet, these results are frequently undocumented, unassigned, or missed entirely, increasing the risk of suboptimal treatment, readmissions, and disease progression. Documentation in discharge summaries is often incomplete, and follow-up responsibilities are frequently unclear. Several interventions have demonstrated success in closing this gap, including EMR-based alerts, pharmacist-led stewardship programs, and auto-populated discharge tools. Systematic reviews suggest that these interventions are most effective when implemented as part of a broader, multidisciplinary strategy that includes clinical teams, pharmacists, discharge planners, and institutional oversight.

Ultimately, discharging a patient with pending cultures is not inherently unsafe, but doing so without a structured follow-up system is. Hospitals and healthcare systems should develop and enforce standardized protocols that clearly document pending results, assign responsibility for follow-up, and ensure that every finalized culture is reviewed and acted upon. Closing this loop is not only feasible but essential for improving patient safety and quality of care during the critical transition out of the hospital.

## References

[1] Roy CL, Poon EG, Karson AS, Ladak-Merchant Z, Johnson RE, Maviglia SM, Gandhi TK (2005). Patient safety concerns arising from test results that return after hospital discharge. Ann Intern Med.

[2] El-Kareh R, Roy C, Brodsky G, Perencevich M, Poon EG (2011). Incidence and predictors of microbiology results returning postdischarge and requiring follow-up. J Hosp Med.

[3] Walz SE (2012). Pending microbiology cultures at hospital discharge and post-hospital patient outcomes in Medicare patients discharged to sub-acute care. https://asset.library.wisc.edu/1711.dl/KMTZXIAQQLW4S87/R/file-83df7.pdf.

[4] Walz SE, Smith M, Cox E, Sattin J, Kind AJ (2011). Pending laboratory tests and the hospital discharge summary in patients discharged to sub-acute care. J Gen Intern Med.

[5] Kantor MA, Evans KH, Shieh L (2015). Pending studies at hospital discharge: a pre-post analysis of an electronic medical record tool to improve communication at hospital discharge. J Gen Intern Med.

[6] Kailani L, Andrews MM, Barns B (2016). Cultures pending at discharge: systematically closing the loop to improve patient safety. Open Forum Infect Dis.

[7] Darragh PJ, Bodley T, Orchanian-Cheff A, Shojania KG, Kwan JL, Cram P (2018). A systematic review of interventions to follow-up test results pending at discharge. J Gen Intern Med.

[8] Whitehead NS, Williams L, Meleth S (2018). Interventions to improve follow-up of laboratory test results pending at discharge: a systematic review. J Hosp Med.

[9] Fay LN, Wolf LM, Brandt KL, DeYoung GR, Anderson AM, Egwuatu NE, Dumkow LE (2019). Pharmacist-led antimicrobial stewardship program in an urgent care setting. Am J Health Syst Pharm.

[10] Van Abel AL, Virk A, Cole K (2024). Impact of pharmacist-led microbiology result follow-up post-discharge for patients undergoing inpatient infectious diseases consultation. Antimicrob Steward Healthc Epidemiol.

[11] Boockvar KS, Carlson LaCorte H, Giambanco V, Fridman B, Siu A (2006). Medication reconciliation for reducing drug-discrepancy adverse events. Am J Geriatr Pharmacother.

